# Ginsenosides Rg5 and Rk1 Enriched Cultured Wild Ginseng Root Extract Bioconversion of *Pediococcus pentosaceus HLJG0702*: Effect on Scopolamine-Induced Memory Dysfunction in Mice

**DOI:** 10.3390/nu11051120

**Published:** 2019-05-20

**Authors:** Kyu Sup An, Yeo Ok Choi, So Min Lee, Hyeon Yeol Ryu, Su Jin Kang, Yong Yeon, Yu Ri Kim, Jae Geun Lee, Chul Joong Kim, Ye ji Lee, Byeong Ju Kang, Jee Eun Choi, Kyung Seuk Song

**Affiliations:** 1 Korea Conformity Laboratories, 8, Gaetbeol-ro 145 beon-gil, Yeonsu-gu, Incheon 21999, Korea; ksahn@kcl.re.kr (K.S.A.); somin14@kcl.re.kr (S.M.L.); rhyckato98@kcl.re.kr (H.Y.R.); kang1973@kcl.re.kr (S.J.K.); dydrkfl28@kcl.re.kr (Y.Y.); 2 Bio Research Institute of Biotechnology, 504 Ilho Golden Tower, 11, Jeongbalsan-ro, Ilsandong-gu, Goyang-si, Gyeonggi-do 10402, Korea; wbio2008@gmail.com; 3 JI Bio pharm Inc., 805 Business Building, Dong Guk University, 32, Dongguk-ro, Ilsandong-gu, Goyang-si, Gyeonggi-do 10326, Korea; yuri@jibio.co.kr (Y.R.K.); qudwn5825@bioribb.com (B.J.K.); bioribb@bioribb.com (J.E.C.); 4 Hwajin BioCosmetics CO., LTD., 303 1 Building, Chuncheon Bioindustry Foundation, 32, Soyanggang-ro, Chuncheon-si, Gangwon-do 24232, Korea; leejeakun@hanmail.net (J.G.L.); kjjup2@naver.com (C.J.K.); yj6013@hanmail.net (Y.j.L.)

**Keywords:** cultured wild ginseng root, *Pediococcus pentosaceus*, ginsenoside Rg5/Rk1, acetylcholinesterase, memory deficit

## Abstract

Wild ginseng is known to contain additional physiologically and pharmacologically active substances than common ginseng. The utilization of this herb can be maximized by altering its composition via tissue culture generating adventitious roots. We enriched the content of specific ginsenosides and investigated their role in ameliorating memory impairment. Cultured wild ginseng root was subjected to extraction, steaming, and fermentation using *Pediococcus pentosaceus HLJG0702* to enhance the levels of ginsenosides Rg5 /Rk1. The analysis of product, HLJG0701, confirmed target ginsenosides. We analyzed the inhibitory effect of ginsenoside Rg5/Rk1, HLJG0701 and the raw material on acetylcholinesterase. Further, we performed Morris water maze, Y-maze, and passive avoidance tasks with mice exhibiting memory deficit induced by scopolamine, and we analyzed the concentrations of acetylcholinesterase and acetylcholine in their brains. Studies showed that the levels of ginsenosides Rg5 /Rk1, not found in the raw material, were enhanced in HLJG0701. Ginsenosides and HLJG0701 significantly inhibited acetylcholinesterase unlike the raw material. In all behavioral tasks, HLJG0701 showed memory improvement. It reduced acetylcholinesterase, whereas, it preserved acetylcholine in brain. In conclusion, cultured wild ginseng root extract fermented by *P. pentosaceus HLJG0702* contains the distinctive ginsenosides Rg5/Rk1, which may ameliorate memory impairment via inhibition of acetylcholinesterase resulting in increased acetylcholine levels in the brain.

## 1. Introduction

Ginsenoside is saponin, the major physiologically active ingredient in ginseng (the root of *Panax ginseng C.A. Meyer*), which not only displays pharmacological activity in the central nervous, endocrine, cardiovascular, and immune systems, but also exhibits anti-inflammatory, antioxidant, and anti-cancer effects [1,2]. Studies have identified more than 30 types of ginsenosides so far and many of them improve memory and learning in neurodegenerative diseases. For example, major ginsenosides such as Rg1, Rb1, Re, and Rd contribute to the prevention of memory deficits via mechanisms including reduction of reactive oxygen species (ROS) nuclear factor kappa-light-chain-enhancer of activated B cells (NF-κB) signaling pathway, nitric oxide (NO) generation, lactate dehydrogenase (LDH) release, β amyloid (Aβ) protein expression, and acetylcholinesterase (AChE) activity [3]. In addition, a few studies showed that minor ginsenosides such as Rg3, Rh2, Rg5, and Rk1 also ameliorate memory impairment [4,5]. However, additional studies are required in order to confirm the pharmacological effects of minor ginsenosides which often have a more powerful pharmaceutical potential than the majors [6].

Wild ginseng is naturalistically germinated ginseng. It is commonly known that wild ginseng contains a diverse range of ginsenosides at a higher concentration than in common ginseng [7]. However, currently, wild ginseng is scarce prompting the need for tissue culture techniques to maximize the utilization of wild ginseng. Techniques such as steaming, puffing, fermentation, and high-temperature/pressure treatments have been used to alter, enhance, or enrich ginsenosides present in ginseng [8]. Minor ginsenosides that are naturally present in trace amounts can be enriched during this transformation. In particular, fermentation techniques using lactic acid bacteria have been found to dramatically increase the contents of ginsenosides Rg5 and Rk1 [6].

In the present study, we established a manufacturing process to enrich the content of ginsenosides Rg5 and Rk1 in the wild ginseng root obtained via tissue culture. The product, fermented extracts of cultured wild ginseng root (HLJG0701), was analyzed using high-performance liquid chromatography (HPLC) to verify the levels of ginsenosides Rg5 and Rk1. Further, we investigated the neuroprotective effects of fermented extracts of cultured wild ginseng root in scopolamine-induced mouse model amnesia.

## 2. Materials and Methods

### 2.1. Animals

Six male C57BL mice (20–23 g) were purchased from (Orient Bio Inc, Gyeonggi-do, Korea). The C57BL mice are known to be the best performers in the Morris water maze task among all the mouse strains [9]. All animals were housed under controlled conditions of temperature 22.5 ± 0.5 °C, relative humidity 47.7 ± 4.7%, lighting intensity 260 Lux, noise 49.6 dB, and 12 h light/dark cycle. Each of the polycarbonate cages housed two mice, which were fed with a laboratory rodent diet (Envigo, USA) and provided with water (treated with reverse osmosis) ad libitum. They were acclimated for six days before initiation of treatment with test materials and only animals with the best appearance were used in this study. All experiments were conducted in compliance with the Guidelines for Laboratory Animal Use and approved by the Institutional Animal Care and Use Committee of the Korea Conformity Laboratories (approval No. IA17-00638). 

### 2.2. Treatments

Animals were distributed according to their graded body weights and randomized into six test groups (eight mice per a group) including the control group, negative group, three HLJG0701 dosing groups, and positive group. Control and negative groups were orally treated distilled water as a vehicle and HLJG0701 dissolved in the vehicle was given to mice at doses of 150, 300, and 600 mg/kg by gavage. Donepezil, as a positive control, was injected intraperitoneally. All test agents were administered once a day during three weeks before initiation of behavioral tests. During behavioral tests, all test agents, except the control group, were administered 1 h before each trial and memory impairment was induced by 1 mg/kg of scopolamine (i.p.) at 30 min after treatment with test agents. Scopolamine hydrobromide and donepezil were purchased from (Sigma Aldrich Inc., St. Louis, MO, USA and Jubilant Generics Ltd., Mysore, Karnataka, India), respectively.

### 2.3. Manufacturing Process of HLJG0701

The cultured wild ginseng root, representing the raw material, was isolated originally from a 35-year old wild ginseng in Yanggu-gun (Gangwon-do, Korea). A series of manufacturing processes were used to produce the fermented extract of cultured wild ginseng root, HLJG0701. The steps involved extraction using 70% fermented alcohol, purification of saponins, vacuum evaporation, suspension, steaming, and fermentation with *Pediococcus pentosaceus HLJG0702*, a type of lactobacillus.

The raw, intermediate product after purification, and the final product (HLJG0701) in each manufacturing step were quantitatively analyzed to determine the levels of contents of the standard ginsenosides Rg5 and Rk1 using an HPLC system (Thermo Ultmate 3000 series), a UV detector (RS variable wavelength detector), and the Capcell pak C18 column (Shiseido, 5 μm, 250 × 4.6 mm). Samples were filtered with 0.45 μm membrane and analyzed with 10 μL of injection volume at 1.0 mL/min flow rate, and 30 °C of column temperature, and detected at 203 nm wavelength. The HPLC conditions were as follows: solvent A, water; solvent B, acetonitrile; gradient, 0–5 min (20%, B), 5–20 min (20–23%, B), 20–25 min (23–30%, B), 25–30 min (30–40%, B), 30–35 min (40–50%, B), 35–60 min (50–85%, B), 60–65 min (85%, B), 65–66 min (85–20%, B), and 66–75 min (20%, B). The standard ginsenosides Rg5 and Rk1 were purchased from Sigma Aldrich Inc. and the lactic acid bacteria were obtained from Hwajin BioCosmetics Co., Ltd. (Gangwon-do, Korea).

### 2.4. AChE Activity Inhibition Assay

The AChE inhibition assay of Rg5, Rk1, the raw material, and HLJG0701 was performed in compliance with the colorimetric method [10]. The reaction mixture and 0.2 U/mL of AChE (Abcam, Cambridge, UK) were added to each well of the microplate containing the test sample, followed by exposure to blocking light for 30 min at room temperature. The absorbance was scanned at 410 nm using a microplate reader (Spectramax PLUS 384, Molecular Device, San Jose, CA, USA). The measured values were corrected based on the absorbance of the reaction mixture and test samples only and the AChE inhibition rate was calculated as a percentage according to the following equation: AChE inhibition rate = 100 − (As/Ac) × 100; As, absorbance of the test sample; Ac, absorbance of the control. A positive control was selected as donepezil.

### 2.5. Morris Water Maze Task

The maze was a stainless steel circular pool made with a diameter of 120 cm and a height of 50 cm. The pool was equally divided into four imaginary quadrants (NW, NE, SE, and SW), corresponding to the visual cues of different shapes (square, triangle, circle, and star) attached to the wall. A transparent escape platform (6 cm in diameter and 29 cm in height) was located in the center of one quadrant (NE) and the pool was filled with water until the platform was submerged 1 cm below the surface. The water was made opaque with titanium oxide and maintained at 22 ± 2 °C. All the experimental conditions were maintained for the duration of the experimental period.

During the two days before trial initiation, all animals were allowed to swim in the pool without a platform daily for 60 s in order to evaluate their locomotor activity and acclimation to the surrounding environmental conditions. Training sessions were conducted with two trials per day at an interval of 120 s for five consecutive days. Mice were semi-randomly released in one quadrant where the platform was not placed and the escape latency, the time taken by the mouse to reach the hidden platform, was measured subsequently. Mice were allowed 60 s for exploration of the platform. It was allowable for them to stay on it for 15 s, once they found the platform. If the mice failed to locate the platform on their own, they were gently guided and allowed to remain on it for 15 s.

The next day after the last training, the probe test was conducted in the pool without the platform. Animals were allowed to swim for 60 s on the opposite quadrant in which the platform was located previously. During that period, the time spent in the target quadrant as well as swimming distance were recorded. The behavior of mice was computed using a video tracking system (EthoVision XT, Noldus, USA).

### 2.6. Y-Maze Task

The Y-maze consisted of three white arms symmetrically connected at an angle of 120° (A, B, and C arm, 35 cm length × 6 cm width × 13 cm height). After a mouse was placed at the distal end of one arm, the sequences (i.e., ABCAC etc.) and the total number of entries into each arm were recorded during 8 min of free movement. The entry was recognized only when the whole body, including the tail fully entered the arm. An actual alteration, which was defined as the combination of successive entries into each arm (i.e., ABC, BCA, CAB. not ACA etc.), was counted and the spontaneous alteration was calculated using the following equation: %alteration = [actual alteration / (total arm entries − 2)] × 100. The number of total arm entries was an indicator of the locomotor activity of the animals.

### 2.7. Passive Avoidance Task

The apparatus for passive avoidance task included two compartment boxes (30 × 30 × 30 cm) connected with each other by a guillotine door. One box was illuminated by a light bulb inside while the other was a dark compartment containing electric rods on the floor. In the acquisition trials, a mouse was placed in the illuminated box and a guillotine door was opened after 10 s. If the animal entered a dark compartment, the door was automatically closed, and the animal received an electric shock with 0.3 mA from the floor for 3 s. If the animal did not enter the dark chamber during the 90 s period, the animal was carefully guided. After 24 h, the retention trials were performed in the same manner as the previously trial, except for the electric shocks. The latency time, which started from the door opening until the mouse entered the dark box, was measured for a maximum of 300 s.

### 2.8. AChE Activity and Contents of ACh in Brain Tissues

Mice were euthanized immediately after the last behavioral test followed by extraction of their brains except for the cerebellum. The tissue was homogenized in 1 mL of ice-cold phosphate-buffered saline (pH 7.4, Gibco) and centrifuged at 5000 rpm/min for 10 min at 4 °C. The supernatant was used to determine the AChE activity and the ACh level. Equal amounts of all samples were analyzed after the protein assay using the Micro Pyrogallol Red method. Measurements were conducted using assay kits for AChE and ACh (Abcam, Cambridge, UK) according to the manufacturer’s instructions. 

### 2.9. Statistical Analysis

All data were presented as means ± SEM. During the Morris water maze task, differences between groups (control vs. negative group, or negative control vs. HLJG0701 groups) were verified using repeated measures analysis of variance (ANOVA). During the others, statistical significance between the control and the negative group was tested by the Student’s t-test. The negative control and the HLJG0701 group were compared by one-way ANOVA to determine significances between the groups as well as the equality of variance. Statistical results were subjected to post-hoc comparison. If the results showed equal variance the Duncan’s test was used, otherwise, the Dunnett’s T3 test was performed. Statistical significance was analyzed using SPSS software (Chicago, IL, U.S.A.) and was only considered if the *p*-value was under 0.05.

## 3. Results

### 3.1. Analysis of HLJG0701

**Figure 1:**
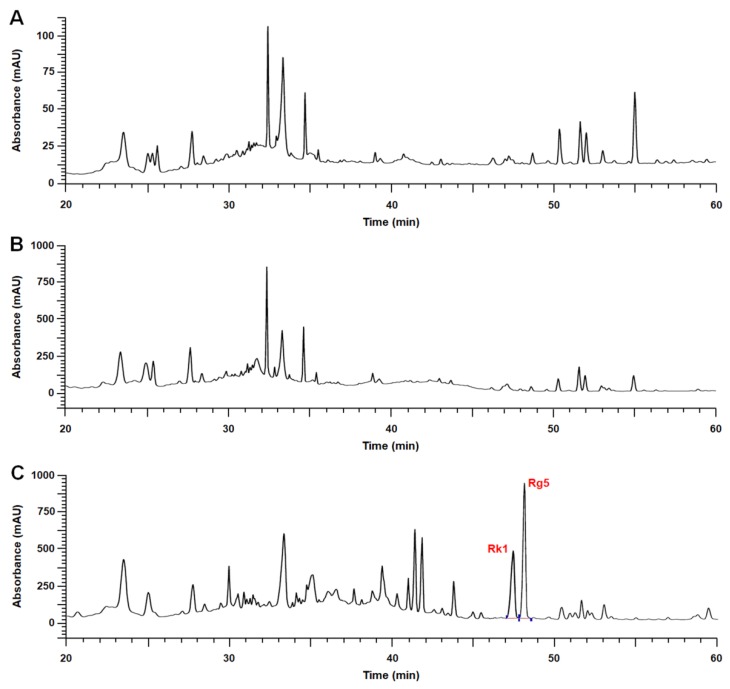
Representative HPLC chromatograms of products analyzed in this study. (**A**) The raw material (cultured wild ginseng root) and (**B**) the intermediate product seldom contained ginsenosides Rg5 and Rk1. (**C**) Concentrations of ginsenosides Rg5 and Rk1 in HLJG0701, the fermented extract of cultured wild ginseng root, were detected as 21.48 and 18.71 mg/g respectively.

Ginsenosides Rg5 and Rk1, the target components of HLJG0701, were almost undetected in the raw and the intermediate products. However, quantitative analysis of HLJG0701 revealed that ginsenosides Rg5 and Rk1 contents were determined at 21.48 and 18.71 mg/g, respectively, in the final product. (Table 1 and Figure 1).

Quantitative analysis included three different lots of HLJG0701, and each lot was measured three times repeatedly.

### 3.2. AChE Activity Inhibition Assay

**Figure 2:**
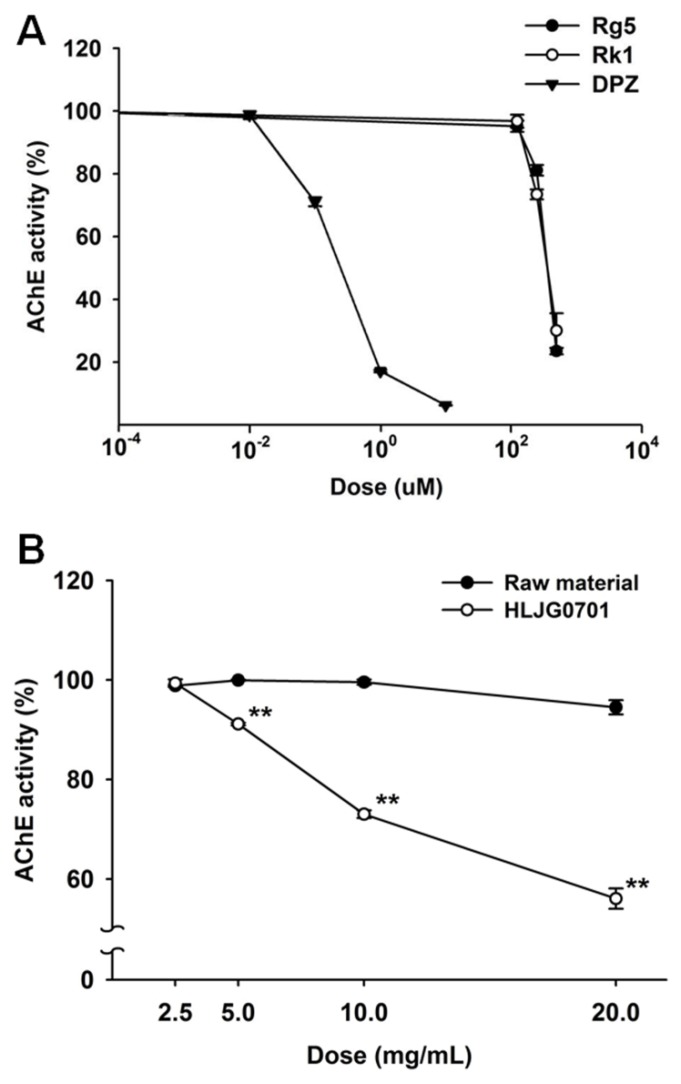
(**A**) Effect of standard ginsenosides Rg5 and Rk1 on AChE activity. The AChE inhibition rate of Rg5, Rk1 and donepezil (DPZ) was analyzed. Donepezil was used as a positive control. (**B**) The AChE inhibition rate of HLJG0701 and its raw material was compared. Data are presented as mean ± SEM (*n* = 3). ^**^ *p* < 0.01 vs. raw material.

We not only measured the AChE inhibition rate of ginsenosides Rg5 and Rk1 but also compared the raw material and HLJG0701 to confirm their role in AChE inhibition. Both Rg5 and Rk1 inhibited AChE similarly in a dose-response correlation, with IC_50_ values of 376 μM and 386 μM, respectively (Figure 2A). However, the activities of the raw material and HLJG0701 showed variation. The HLJG0701 form significantly inhibited AChE activity under increased concentration, unlike the raw material with undetectable Rg5 and Rk1 (Figure 2B).

### 3.3. Morris Water Maze Task

**Figure 3:**
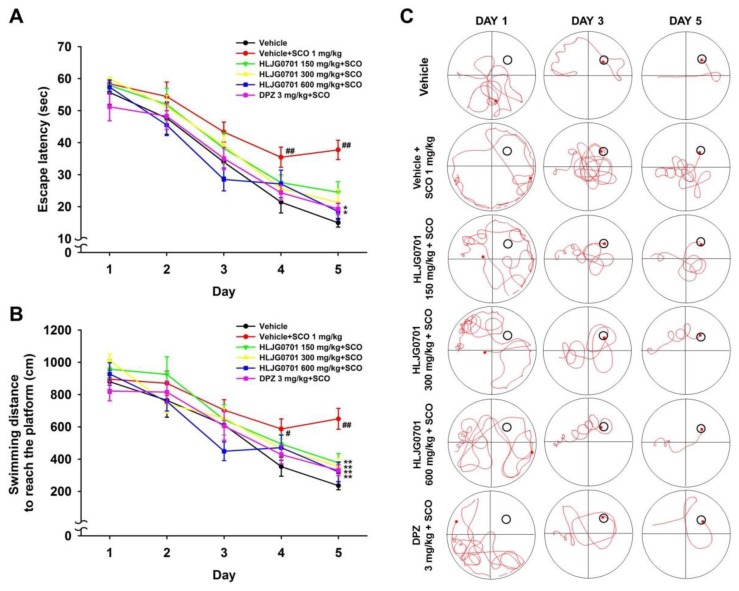
Effect of HLJG0701 on memory deficit in training sessions involving the Morris water maze task. To evaluate the effect of memory improvement, (**A**) the escape latency and (**B**) swimming distance to reach the platform were measured in training sessions during 5 consecutive days. Vehicle (control and negative groups, p.o.), HLJG0701 (p.o.), and donepezil (DPZ, positive group, i.p.) were treated and memory impairment was induced by scopolamine (SCO, i.p.). Data are expressed as mean ± SEM (*n* = 8). ^#^ *p* < 0.05, ^##^ *p* < 0.01 vs. control group, ^*^ *p* < 0.05, ^**^ *p* < 0.01 vs. negative group. (**C**) Representative images tracking the swimming movement on day 1, 3, and 5 in training sessions. The red curve tracks the movement of mice and the last location of mice is marked with the red square. The black circle indicates an escape platform.

**Figure 4:**
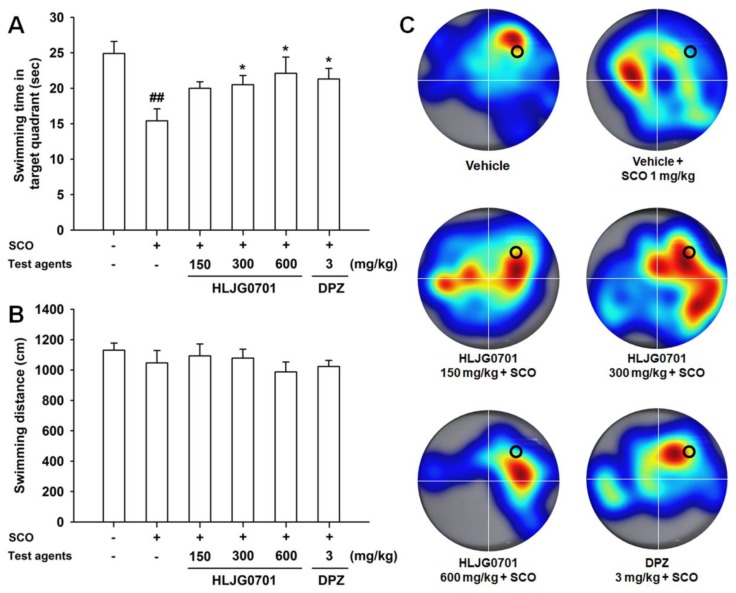
Effect of HLJG0701 on reference memory in probe trial sessions of the Morris water maze task. Mice were allowed to swim in the water tank without a platform for 60 s. (**A**) Time spent in the quadrant where the platform was previously located was recorded, and (**B**) the total swimming distance also was measured. Treatment with test agents was the same as described in Figure 4. Data are expressed as mean ± SEM (*n* = 8). ^##^ *p* < 0.01 vs. control group, ^*^ *p* < 0.05 vs. negative group. (**C**) Representative heatmap images of probe trial sessions. The longer the mice spent in each quadrant, the redder is the image displayed. Conversely, a cooler color indicates a shorter duration. The black circle shows the position where the platform was located previously.

The Morris water maze is an effective tool for the evaluation of hippocampus-dependent spatial learning and reference memory [11]. Measurement of time to reach the platform revealed that the escape latency tended to decrease during the training session in all test groups. However, latency of each group varied with time. The escape latency in the negative group was significantly prolonged on day four and five as compared with that of the control group (*p* < 0.01). By contrast, the HLJG0701 concentration of 600 mg/kg and donepezil significantly shortened the escape latency on day five as compared with the negative group (*p* < 0.05). Dose levels below 300 mg/kg also decreased the escape latency but there was no statistical significance (Figure 3A). With respect to the swimming distance to reach the platform, similar results were observed. The mice in the HLJG0701 150, 300, and 600 mg/kg as well as donepezil groups moved significantly shorter distances until reaching the platform on day five as compared with the animals in the negative group (*p* < 0.01). In addition, the mice in the negative group swam a significantly longer distance to the destination on day four (*p*< 0.05) and day five (*p* < 0.01) as compared with that of the control group (Figure 3B). In the probe test on the following day, the time spent in the platform quadrant of the negative group was significantly decreased, as compared with that of the control group (*p* < 0.01). On the other hand, the swimming time was extended in groups treated with more than 300 mg/kg of HLJG0701 and donepezil (*p* < 0.05). Although there was no statistical significance, increased swimming time was observed in mice exposed to the HLJG0701 150 mg/kg (Figure 4A). Meanwhile, the foregoing results were not affected by the locomotor activity of animals because the effect of swimming distances was not statistically significant in the different groups (Figure 4B).

### 3.4. Y-Maze Task

**Figure 5:**
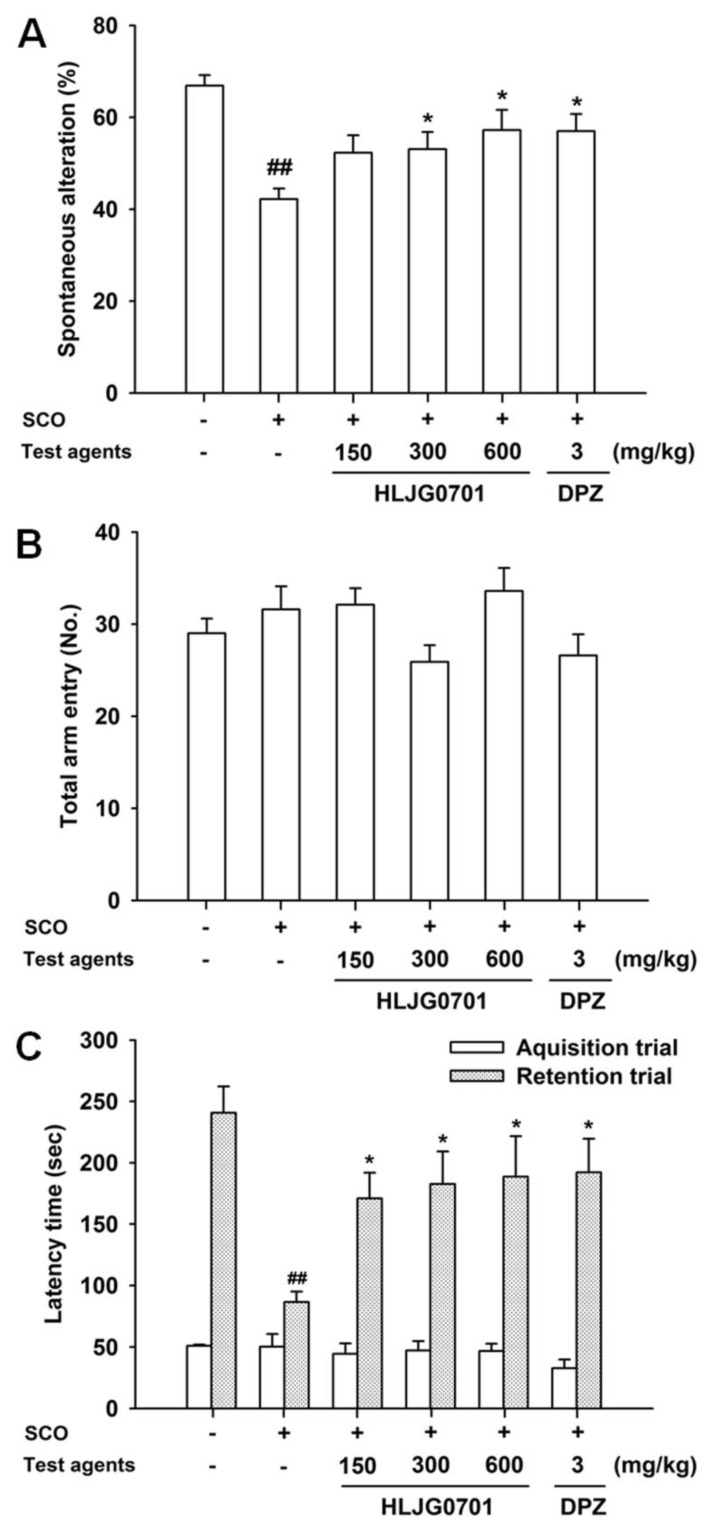
Effect of HLJG0701 on spatial and learning memory in memory deficient mice. (**A**) Spontaneous alteration and (**B**) total arm entry in Y-maze task as well as (**C**) latency time in passive avoidance task were investigated. Vehicle (control and negative groups, p.o.), HLJG0701 (p.o.), and donepezil (DPZ, positive group, i.p.) were given 1 h before trials and scopolamine (SCO, i.p.) was injected 30 min after treatment to induce memory deficit. Values are presented as mean ± SEM (*n* = 8). ^##^ *p* < 0.01 vs. control group, ^*^ *p* < 0.05 vs. negative group.

Spatial working memory can be assessed using the Y-maze task [12]. Spontaneous alteration was significantly lower in the negative group than in the control group (*p* < 0.01). It was significantly elevated in the HLJG0701 groups exposed to above 300 mg/kg and donepezil, compared with the negative group (*p* < 0.05, Figure 5A). However, the calculated percentage of alteration in the HLJG0701 300 mg/kg group did not differ dramatically from the value of the negative group. In the HLJG0701 concentration of 600 mg/kg and donepezil groups, spontaneous alteration was only relatively higher than the negative group. It was concluded that the results were caused by treatment and not the locomotive abilities of the animals since the total arm entries did not show any significant differences (Figure 5B).

### 3.5. Passive Avoidance Task

This is a fear-motivated task which has been employed to assess learning and memory [13]. In retention trials, a significant decrease was found in the latency time of the negative group, as compared with the control group (*p* < 0.01), whereas, HLJG0701 significantly prolonged the latency time in a dose-dependent manner in all dose levels. Donepezil also increased the latency time, which showed the optimal effect (*p* < 0.05, Figure 5C). We found no significant differences in the latency time between groups in the acquisition trials. 

### 3.6. AChE Activity and ACh Contents in Brain Tissues

**Figure 6:**
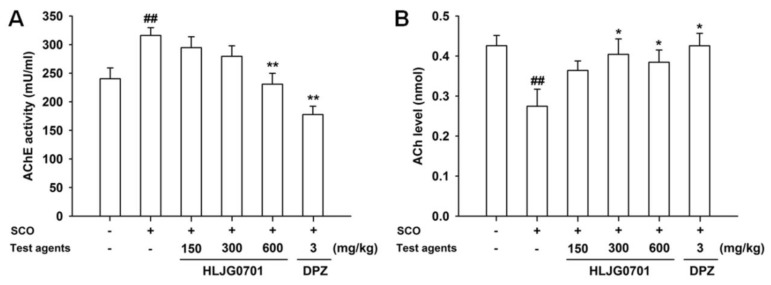
Effect of HLJG0701 on AChE and ACh in the brains of memory-deficient mice. (**A**) AChE activity and (**B**) ACh levels were measured in the brain extracts (except for cerebellum) from animals immediately after termination of the last behavioral test. Vehicle (control and negative groups, p.o.), HLJG0701 (p.o.), and donepezil (DPZ, positive group, i.p.) were used for treatment and memory impairment was induced by scopolamine (SCO, i.p.). Data are presented as mean ± SEM (*n* = 8). ^##^ *p* < 0.01 vs. control group, ^*^ *p* < 0.05, ^**^ *p* < 0.01 vs. negative group.

AChE activity and ACh contents were determined in the mouse brain except for the cerebellum. The AChE activity in the negative group was significantly higher than in the control group (*p* < 0.01). Exposure to HLJG0701 600 mg/kg and donepezil significantly reduced AChE activity, compared with the activity of the negative group (*p* < 0.01). Only the HLJG0701 600 mg/kg group showed a significant difference, however, AChE activity was decreased in a dose-dependent manner in all HLJG0701-treated groups (Figure 6A). On the other hand, the ACh content showed the opposite trend. Scopolamine treatment resulted in a decline in the ACh level in the negative group as compared with the control group, however, treatment with HLJG0701 and donepezil preserved higher ACh contents than that of the negative group. Moreover, significant differences were observed in the negative group (vs. control group, *p* < 0.01) as well as the groups exposed to HLJG0701 300 and 600 mg/kg and donepezil (vs. negative group, *p* < 0.05, Figure 6B).

## 4. Discussion

Ginsenosides have the triterpenoid dammarane, with a distinguishing aglycone skeleton structure, which is not found in other plants species except for ginseng [12]. Ginsenosides in the dammarane families are classified as protopanaxadiol and protopanaxatriol groups based on the number of hydroxyl groups [14]. It is generally believed that ginsenosides occur in wild ginseng more than in common ginseng. Despite of the advantage, wild ginseng is not widely used because it is not available naturally, unfortunately. This limitation can be overcome by culturing the adventitious roots obtained from the tissues of wild ginseng, which has been known to show fewer differences in genomic DNA or content of saponin content as compared with the wild species [15]. As compared with the major ginsenosides (Rb1, Rg1, Rc, Rd, Re, etc.) found abundantly in this herb, minor ginsenosides exist in very low quantities or are even absent [6]. However, high levels of ginsenosides Rg5 and Rk1 were detected in the fermented extract of cultured wild ginseng root (HLJG0701), with contents reaching 21.48 and 18.71 mg/g, respectively, in HPLC analysis. It is possible that steaming altered the structure of ginsenosides to a low polarity via hydrolysis and dehydration, resulting in the synthesis of new ginsenosides. Hydrolysis may have transformed the Rb1 and Rc in the protopanaxadiol group into Rg3 by removing the glycosyl moiety attached to C-20 followed by conversion into Rg5/Rk1 via dehydration [16]. A recent study has found that organic acids convert ginsenoside Rb1 to Rg3 and Rg5 [17]. *P. pentosaceus*, a type of lactic acid bacteria, increases organic acids that create acidic environments (low pH and high acidity). Under these conditions, deglycosylation of ginsenoside is facilitated by acidic hydrolysis [6]. The foregoing studies support our synthetic approach in this study, considering that standard components of the product, Rg5/Rk1, were scarcely detected in the raw cultured root, but were greatly enhanced after steaming and fermentation by *P. pentosaceus HLJG0702*.

The effects of ginsenosides on brain have been demonstrated at the level of molecular mechanism on neuronal cells and neurotransmission. In astrocytes, ginsenoside elevated the expression of glutamate transporter-1 (GLT-1) and the level of phosphorylated protein kinase B (PKB/Akt). It also reported that upregulation of antioxidant systems such as glutathione S-transferase (GST) and hemeoxygenase-1 (HO-1) by ginsenoside resulted in reducing cell death induced by H_2_O_2_ in rat cortical astrocytes [18]. Microglia are immune cells in the central nervous system. They are activated to defense the brain and support neuronal cell survival. At the same time, overactive microglia can cause neuroinflammation due to the release of inflammatory mediators including NO, ROS, and cytokines. Ginsenosides not only decrease proinflammatory cytokines such as tumor necrosis factor-α, interleukin (IL)-1β, IL-6, inducible nitric oxide synthase (iNOS), and cyclooxygenase-2 (COX-2), but they increase expression of HO-1 via the nuclear factor erythroid 2-related factor 2 (Nrf2) pathway resulting in reduce ROS and NO levels in microglia [19,20]. Ginsenosides also affect neurotransmission by a variety of mechanisms that include the regulation of synthetic enzyme, the signaling pathways in specific neurotransmitter systems, and release of neurotransmitter [21]. Among the neurotransmitters, particular neurotransmitters are distinctively involved in memory formation and recovery. Glutamate, GABA, dopamine, and acetylcholine have been known to influence stronger impact on cognitive function than serotonin and norepinephrine [22]. In addition, since the association between cholinergic dysfunction and age has been studied for the first time in memory impairment [23], many studies have reported that the change in cholinergic function was most strongly correlated with the decline of cognitive function [24,25]. Acetylcholine is the major neurotransmitter in the central cholinergic pathway and is hydrolyzed by AChE [26]. In the present study, several samples were investigated for inhibition of AChE activity. Our results showed that Rg5/Rk1 suppressed AChE activity. Furthermore, a few assays were conducted to compare the cultured wild ginseng root and its fermented form, HLJG0701, and interestingly only the fermented form showed a significant inhibition of AChE activity.

We also performed behavioral tests in mice with memory deficits induced by scopolamine. Scopolamine, a non-selective muscarinic acetylcholine receptor antagonist, disrupts learning and short-term memory [27]. Behavioral tests performed in this study are well known as effective methods for evaluation of short-term or reference memory based on spatial learning and fear motivation [11,13,28]. On the basis of previous studies, memory improvement was measured according to the results in each of the following tests: reduced latency escape, shortened distance to reach the platform and prolonged swimming time in the target quadrant of the Morris water maze task; increased spontaneous alteration in the Y-maze task; and extended latency time in the passive avoidance task. Since our test results with HLJG0701 using mice exhibiting scopolamine-induced memory loss complied with the above indicators to confirm memory improvement, it suggests that HLJG0701 significantly alleviated memory impairment. Scopolamine also acts particularly in the hippocampus and increases AChE activity in that organ [29]. We found that HLJG0701 suppressed AChE activity but elevated ACh levels in cerebral tissue extracted from the mice.

On the basis of all the foregoing results, it can be concluded that the increased levels of Rg5/Rk1 in the fermented extract of cultured wild ginseng root inhibit the activity of AChE raising the ACh content. Therefore, based on this mechanism, HLJG0701 improved memory dysfunction in scopolamine-induced mouse model of amnesia. 

With aging, a number of neurotransmitter systems such as glutamine, histamine, dopamine, and choline appear to be compromised in humans [30]. In addition, scopolamine induced memory deficit in healthy young humans paralleled the pattern of impairment found in non-demented elderly [31]. On the basis of our results which established the effects of HLJG0701 to include anti-AChE activity, preservation of ACh level, and neuroprotection against scopolamine, it is possible that HLJG0701 ameliorated the decline in cognitive function associated with aging.

Meanwhile, the memory impairment induced by scopolamine is non-degenerative [5], and in the mechanism differs from neurodegenerative diseases such as Alzheimer’s disease (AD). Many studies have reported pathological hypothesis for the onset of AD. Aβ are derived from amyloid precursor protein (APP) by enzymatic cleavage and they accumulate excessively in the brain producing amyloid plaque, and they are also involved in the hyper-phosphorylation and acetalization of tau. These eventually result in neurofibrillary tangles, synapse loss, and neuronal cell death, which it is widely posited to be the cause of AD-related pathology [32]. Recent genome-wide association studies revealed that apolipoprotein (apo) E4 is the significant gene related with age-associated cognitive decline in humans and it was proven as a genetic risk factor for AD [33,34]. Interestingly, ApoE4 is also thought to be a risk factor for diabetes. There are results that hyperglycemia or impaired insulin signaling may be directly involved with the pathophysiology of AD (e.g., tau cleavage and apoptosis) and many reports support that metabolic dysfunction exacerbate the progression of AD in patients [35]. Heavy metals such as lead, mercury, and cadmium ultimately accumulate Aβ aggregate through oxidative stress, dysfunction of tubulin, and changes in specific protein expression (e.g., overexpression of ACHE-S and downregulation of ACHE-R7 by blocking M1 receptor) [36]. Some evidences suggest that even AChE is a pathogenesis of AD. Cholinergic neurons are mainly affected in AD, and its alteration is closely related to the memory and attention deficits observed in AD patients. The structural motif of AChE is found to facilitate the formation of amyloid fibrils [37]. The effect of HLJG0701 in neurodegenerative diseases was mediated via a cholinergic system in this study. Even though we did not investigate the effect on other pathogenesis of AD, HLJG0701 may work effectively based on subsequent studies. Rg5 improved cognitive dysfunction in an AD rat model by attenuating Aβ accumulation and increasing the expression of neurotrophic factors such as brain-derived neurotrophic factor (BDNF) and insulin-like growth factor 1 (IGF-1) [38]. Rk1 exhibits anti-oxidant effects [14]. Peroxisome proliferator activated receptors (PPAR) is a major transcriptional factor in adipogenic differentiation, and inhibition of this process is important in controlling adipogenesis. Ginsenosides Rg5/Rk1 reduced intracellular lipid accumulation by interacting with PPAR, resulting in inhibition of adipogenesis [39,40]. It suggests that this can be effective in diabetes associated with obesity, one of the AD’s incidence factors. Therefore, HLJG0701 is enriched in Rg5/Rk1, which may improve memory deficits in neurodegenerative diseases. Moreover, the effects of HLJG0701 paralleled in the mechanisms and efficacy when compared to other natural compounds studied as potential therapeutic agents for AD. Many natural compounds, such as Silymarin, green tea seed oil, curcumin, and *Cuscuta chinensis*, all showed the protection of cholinergic function including AChE inhibition, and some of them were also found to be effective in reducing oxidative stress and neuroinflammation. The potential neuroprotection functions of HLJG0701 and the similarity with foregoing natural compounds in physiological actions further support its availability as a treatment of neurodegenerative diseases [41,42,43,44].

## 5. Conclusions

Ginsenosides Rg5 and Rk1 were enriched in the cultured wild ginseng root via steaming and fermentation using lactic acid bacteria *P. pentosaceus HLJG0702*. The ginsenoside Rg5/Rk1 not only inhibited AChE activity but also maintained ACh levels. The fermented extract of cultured wild ginseng root, HLJG0701, alleviates memory impairment by regulating cholinergic dysfunction. Therefore, HLJG0701 can be used as a functional food to help improve memory impairment in old age. It may also be effective in neurodegenerative diseases such as AD. The dementia prevalence in Korea is 9.6 people per 1000 population at present, lower than the US (11.6) and the OECD average (14.8). However, Korea will see prevalence more than double in the next 20 years as a most rapidly ageing country (estimated 23.8 people in Korea vs. 23.1 in OECD average) [45]. The potential possibility that HLJG0701 may play an important role in Korea in the future as an AD treatment can be expected, however, further studies are needed to corroborate our findings.

## Figures and Tables

**Table 1:** Quantitative analysis of ginsenosides Rg5 and Rk1 in HLJG0701 using HPLC.

Sample	Contents (mg/g)			
	Lot #1	Lot #2	Lot #3	Average
Ginsenoside Rg5	21.66 ± 0.13	21.63 ± 0.08	21.15 ± 0.22	21.48 ± 0.29
Ginsenoside Rk1	18.89 ± 0.17	18.85 ± 0.09	18.39 ± 0.22	18.71 ± 0.28

## Abbreviations

AChE: acetylcholinesterase, ACh: acetylcholine, ROS: reactive oxygen species, NF-κB: nuclear factor kappa-light-chain-enhancer of activated B cells, NO: nitric oxide; LDH: lactate dehydrogenase, Aβ: β amyloid, HPLC: high-performance liquid chromatography, GLT-1: glutamate transporter-1, PKB: phosphorylated protein kinase B, HO-1: hemeoxygenase-1, GST: glutathione S-transferase, TNF-α: tumor necrosis factor-α, IL: interleukin, iNOS: inducible nitric oxide synthase, COX-2: cyclooxygenase-2, Nrf2: nuclear factor erythroid 2-related factor 2, AD: Alzheimer’s disease, APP: amyloid precursor protein, apo: apolipoprotein, BDNF: brain-derived neurotrophic factor, IGF-1: insulin-like growth factor 1, PPAR: peroxisome proliferator activated receptors.
